# MediaPipe-Based Activity Analysis by Healthcare Professionals: Method Development and Technology Acceptance—A Pilot Study

**DOI:** 10.3390/s26175681

**Published:** 2026-09-07

**Authors:** Tomoko Funayama, Yasutaka Uchida, Yasuhito Asano, Daisaku Soma

**Affiliations:** 1 Department of Occupational Therapy, Teikyo University of Science, Yamanashi 409-0193, Japan; 2 Department of Life & Health Sciences, Teikyo University of Science, Tokyo 120-0045, Japan; 3 Faculty of Information Networking for Innovation and Design, Toyo University, Tokyo 115-8650, Japan; yasuhito.asano@iniad.org; 4 Department of Rehabilitation, Isogo Central Hospital, Kanagawa 235-0016, Japan; rifles060920@gmail.com

**Keywords:** MediaPipe, motion analysis, gait analysis, eating motion analysis, activity assessment, mHealth, technology acceptance, UTAUT2, healthcare

## Abstract

**Highlights:**

**What are the main findings?**

- Healthcare professionals have designed a MediaPipe-based motion analysis method focusing on clinically relevant movements.
- This method enables a simple analysis of gait and eating tasks using single-camera frontal-plane videos that capture key kinematic features from a limited set of variables.

**What are the implications of the main findings?**

- This approach demonstrates the potential for low-cost and potentially applicable motion analyses in clinical and daily settings.
- Effective implementation requires usability improvements and supportive frameworks, including professional training and organizational support.

**Abstract:**

Integrating digital assessment technologies into clinical practice remains a challenge because of usability issues, time constraints, and difficulties in data interpretation. This study aimed to examine the feasibility of a clinically practical MediaPipe-based motion analysis method and to evaluate its technological acceptance by healthcare professionals. We tested an analysis method using single-camera frontal-plane videos of gait, eating, and table-slide tasks to quantify movement under both normal and simulated impairment conditions. To evaluate the clinical utility, a questionnaire based on the unified theory of acceptance and use of technology 2 (UTAUT2) was administered to physical therapists, occupational therapists, and nurses. The results provide preliminary evidence of the feasibility and technology acceptance of MediaPipe for activity analysis by healthcare professionals, even with a limited set of variables. Participants generally showed positive attitudes toward its clinical usefulness; however, challenges related to its usability were identified. These findings also suggest that specialized personnel capable of handling digital technologies may play an important role in facilitating their implementation. With further advances in digital technology, activity assessments are expected to extend beyond clinical settings to everyday environments. To achieve this, not only technological improvements, such as enhanced reliability, validity, and usability, but also comprehensive frameworks incorporating education and organizational support are important.

## 1. Introduction

Recently, the development and widespread adoption of “mobile health (mHealth),” including wearable devices and applications capable of easily and objectively measuring physical activity, have progressed. The use of mHealth is expected to bring about significant transformations in conventional delivery systems by enabling measurements in environments without a therapist, advancing telemedicine, and improving access to healthcare services. Studies applying digital technologies to the healthcare field are also being actively conducted, including the use of patient-generated health data obtained via wearable devices and smartphones [1,2,3,4,5]. Smartphones are ubiquitous and equipped with various sensors. By combining these sensors with AI-driven analysis, the potential for clinical applications can be expanded. In addition, recent studies have emphasized that reliable clinical implementation of digital assessment tools also depends on robust system-level design, including reliable data acquisition, hardware–software integration, and experimental validation [6,7,8]. However, although high expectations have been placed on these digital health technologies, their practical applications in healthcare services remain limited [9,10]. Although these technologies have been recognized by healthcare professionals for their utility, they are not sufficiently utilized in clinical settings [11,12]. However, several barriers to their adoption have been identified. First, concerns exist regarding the reliability and accuracy of patient-generated data [13,14,15,16,17].

A critical issue is the increased workload associated with the growing volume of collected data and the difficulty of converting these data into clinically meaningful information. In clinical practice, clear and intuitive indicators are essential, including speed, comfort, and stability [18,19,20]. However, an excessive volume of data can hinder clinical interpretation. A previous study [18] suggested that clinically useful metrics should be limited to 1–3 items for effective visualization. Furthermore, the study indicated that, within routine clinical activities, such as patient care and therapeutic exercises, the setup time should be within one minute and post-measurement processing should be completed within five minutes. Intuitive visualization and immediate feedback, which enable a rapid understanding of the necessary information, enhance the utility and acceptance of the technology [21].

Other challenges include a lack of integration with electronic health records and incompatibility with existing clinical workflows. Additionally, ethical and legal privacy concerns, along with healthcare-system-level issues, such as inadequate reimbursement structures and a lack of established guidelines, have been identified [22,23]. Furthermore, limited digital literacy among users and insufficient education and training, combined with psychological resistance, play a significant role [17,23,24,25,26]. At the organizational and societal levels, additional challenges include the development of clinical guidelines, adaptation to clinical workflows, and the establishment of reimbursement and insurance systems [23,27,28,29,30,31,32]. A distinct characteristic of technology acceptance in healthcare practice is its perceived benefit to others [33]. Consequently, previous studies have employed the technology acceptance model and the unified theory of acceptance and use of technology (UTAUT) to examine this aspect [34,35,36].

In the utilization of digital devices in healthcare, measurements in daily living environments are considered crucial because of the discrepancy between clinical evaluations and real-life settings [5,29,37]. Digital technology has the potential to improve accessibility and enable continuous monitoring in daily life. It also contributes to understanding disease progression and behavioral patterns and supports personalized medicine and self-management [13,14,24,30]. Against this background, efforts to measure the activities of daily living using digital devices are advancing. Walking, as a fundamental indicator reflecting health status, has been extensively studied, with the evaluation of speed, stride length, and cadence using smartphones and wearable sensors [38,39,40]. Progress has also been made in disease identification through machine learning [41] and advanced analyses using multiple sensors or smart insoles [42,43,44]. The validity of these technologies has been demonstrated in step counting during the early post-operative period [45] and tracking symptomatic changes in patients with stroke or Parkinson’s disease [46,47]. Furthermore, the emergence of non-contact, low-cost methods using GPS or single cameras [48,49,50] is shifting the measurement environment from clinical facilities to daily living settings [51]. However, the variability between methodologies and ensuring reliability in real-life environments remain persistent challenges [40,48]. With regard to eating behavior, objective evaluation methods using sensors and image analysis are also being developed. In particular, the “hand-to-mouth movement” serves as a key behavioral feature [52,53], and automated methods for measuring “bite counts” (the frequency of bringing food to the mouth) have been reported [54,55,56,57]. These methods are particularly useful for identifying “eating difficulties” observed in elderly individuals with dementia, such as difficulty starting a meal, stuffing food, or refusal to eat [58,59,60,61]. Other metrics, such as chewing frequency and eating speed, were also utilized [62,63,64]. Although monitoring and intervention through digital technology [65,66] are important for enhancing the quality of care, challenges, such as accuracy in real-life settings, privacy, and integration into clinical workflows, have been noted [52].

Based on these considerations, MediaPipe-based analytical methods for walking and eating, incorporating key perspectives identified by occupational and physical therapists based on their clinical experiences, were tested, and a survey of technology acceptance among healthcare professionals was conducted. MediaPipe is a versatile framework capable of the real-time extraction of joint centers and facial features using a single camera [67,68]. As a low-cost, markerless technology, it has a low barrier to implementation in healthcare settings [69,70]. Clinically, its utility has been demonstrated in gait analysis [71,72], and it is expected to enable the noncontact quantification of clinical indicators, such as compensatory movements during drinking in stroke patients [73].

From a clinical perspective, there is a strong demand for simple and accessible methods that can be applied daily in rehabilitation and other healthcare fields. This study was initiated from a clinical standpoint to address the practical challenges faced in daily rehabilitation settings. The measurements were performed using a single camera in the frontal plane. In addition to walking and eating, an example application of a table-slide analysis was presented, and a questionnaire survey based on the UTAUT2 was conducted among physical therapists, occupational therapists, and nurses.

## 2. Methods

This study comprises two main components. First, occupational and physical therapists designed a MediaPipe-based motion analysis method. Subsequently, MediaPipe was introduced to healthcare professionals, and hands-on experience in the analysis of gait and eating tasks was provided to some participants. Technology acceptance was evaluated using a questionnaire based on UTAUT2. This study adopted a mixed-methods approach, combining method development and questionnaire-based evaluation.

### 2.1. Data Acquisition

The tasks were recorded in the frontal plane using a single camera on a standard mobile device. Specifically, an iPad (9th generation; Apple Inc., Cupertino, CA, USA) was used for the gait tasks, whereas an Android smartphone was used for the eating and table-sliding tasks (Galaxy S24 SC-51E, Samsung Electronics Co., Ltd., Suwon, Republic of Korea, for the eating task; AQUOS sense6 SH-RM19, Sharp Corporation, Sakai, Osaka, Japan, for the table-sliding task). The frontal-plane configuration was selected because it is feasible for routine use in rehabilitation practice and enables easy self-recording in daily life. The recording conditions and procedures were determined and implemented by the physical or occupational therapists. For two recordings, another individual only pressed the recording button under the therapist’s instructions. A physical therapist performed all gait tasks, whereas an occupational therapist performed all eating and table-slide tasks. The participants were rehabilitation professionals, not patients. The dataset comprised three gait tasks (normal walking, simulated right-lower-limb impairment, and simulated right hemiparetic gait), two eating tasks (normal and simulated impairment), and two table-slide tasks (normal and simulated impairment). The simulated eating conditions were characterized by left lateral trunk flexion, reduced hand-movement speed, and a slower eating rhythm. The table-slide task consisted of sliding a towel forward diagonally with the right upper limb; the impaired condition intentionally incorporated reduced elbow extension strength and emphasized the compensatory proximal movements of the trunk and scapula. For the gait tasks, the participants walked toward the camera. Segments in which the body parts moved out of the frame, typically at the end of each recording, were excluded from the analysis. The eating and table-slide tasks captured only the upper body above the table, with the lower body excluded from the video. The durations of the videos used were approximately 3–10 s for the gait tasks and 30–40 s for the eating tasks. When the gait was recorded from the frontal plane, the participant walked toward the camera, causing the proportion of the body occupying the frame to gradually increase as the task progressed. Consequently, the metrics based on body coordinates or distances tended to increase over time. In contrast, sagittal-plane recordings generally maintain a more constant distance between the camera and the subject, making changes in frame occupancy less likely to occur and enabling easier gait analysis compared with frontal-plane recordings. Furthermore, although using multiple cameras can improve measurement accuracy, sagittal-plane recording requires a larger space and carries the risk that other individuals in the room may unintentionally appear in the footage. Therefore, we intentionally selected frontal-plane recordings using a single mobile device to prioritize usability and feasibility in real-world rehabilitation settings. In everyday situations, photographs and videos are typically captured from the frontal plane, and we believe that this approach is also relevant to rehabilitation settings. Based on this usability perspective, frontal-plane recordings were adopted for this study.

### 2.2. Pose Estimation Using MediaPipe

Body joint coordinates were extracted from each video using MediaPipePose (Google, LLC, Mountain View, CA, USA). This model estimates 33 anatomical keypoints per frame, including those for the head, trunk, upper limbs, pelvis, and lower limbs. As each of the 33 keypoints provided values for the X-, Y-, and Z-axes, 99 data elements were obtained for every timestamp. Motion analysis methods were developed using Python 3.8.20 and the MediaPipe library (version 0.10.10) in a Jupyter Notebook environment. The motion analysis procedures were developed collaboratively by occupational and physical therapists. The MediaPipe-based analyses were performed by an occupational therapist, and the resulting data and graphical outputs were reviewed by both occupational and physical therapists. For the hands-on experience, Google Colab was used to enable online sharing, and the participants executed the Python code with MediaPipe (version 0.10.14). MediaPipe was selected because it enables markerless motion analysis using standard video recordings without specialized motion-capture equipment and can be readily implemented in an online environment using Google Colab. These characteristics were consistent with the accessibility and feasibility objectives of the present study. In the subsequent analysis of the gait and eating tasks, only the Y-axis data were used to focus on the vertical movements relevant to the analysis.

#### 2.2.1. Gait Analysis Methods

The gait parameters used in this study were selected to demonstrate the feasibility of extracting clinically interpretable movement characteristics using MediaPipe under simulated conditions, rather than to provide a comprehensive clinical assessment for a specific disease. Gait was recorded in the frontal plane using a single camera. During recording, the participant walked toward the camera, and the relative position of the participant within the frame changed over time. Consequently, the proportion of the body within the image frame gradually increased. This change in scale can affect the magnitude of the estimated joint coordinates. To reduce the influence of this scale variation and to enable a consistent comparison of joint positions across frames, the joint coordinates corresponding to the MediaPipe Y-axis were normalized using the vertical distance between the shoulder and hip joints as a scaling factor. Only the approaching phase of walking was analyzed. The recordings of the participants walking away from the camera were not included, and the participants’ backs were not captured. Using the MediaPipe Y-axis coordinates of the hip, knee, and heel joints, the knee and heel heights and stride times were calculated.

- Scale Normalization

The vertical (y-axis) coordinates were normalized by dividing them by the vertical difference between the shoulder and hip joints at the same time point. For the left lower limb, normalization was performed using the vertical difference between the left shoulder and hip, whereas for the right lower limb, the vertical difference between the right shoulder and hip was used. This normalization method assumes that the vertical distance between the shoulder and the hip remains relatively constant. Therefore, when strong lateral trunk flexion occurs and the shoulder–hip distance varies, the accuracy of the normalized values may decrease.

- Knee and Heel Elevation

The vertical positions of the knee and heel were calculated based on the Y-axis differences between the hip and knee joints, and between the hip and heel joints, respectively. These differences were interpreted as vertical distances from the hip joint. Using the hip joint as a reference point, smaller differences between the hip and knee or heel were interpreted as indicating that the knee or heel was positioned higher. Time-series changes in these Y-axis values were visualized as graphs representing the elevations of the knee and heel. However, as smaller values correspond to higher positions in this representation, which is contrary to the intuitive interpretation of height, additional plots with inverted Y-axes were generated.

- Stride Time

The stride time was defined as the interval between consecutive peaks, representing the lowest vertical positions of the knee and heel. These peaks were extracted from the downward Y-axis signals after smoothing the data using a moving-average filter. To reduce noise in the joint trajectories, a moving-average filter with a window size of five frames was applied to the normalized vertical difference signals. With a frame rate of 30 fps, this window size corresponds to approximately 0.17 s. Peak values were then extracted from the smoothed signals. Differences between consecutive samples were calculated, and peaks were detected based on sign changes. A candidate peak was identified when the difference changed from positive to negative. A neighborhood-based filtering step was applied to minimize the false detections caused by small fluctuations. In this step, a peak was selected only if its value was greater than or equal to those of neighboring samples within a ±0.3-s window, representing the local maximum within this range. The time interval between consecutive peaks was calculated and regarded as the stride time.

#### 2.2.2. Eating Motion Analysis

Two eating conditions were used: normal eating and slow eating with lateral trunk flexion. In both conditions, the participants ate using chopsticks with their right hands. In the slow eating condition, the task included drinking water while holding a cup with the left hand. The eating task was recorded using a camera placed on the frontal plane. The analysis focused on the upper-body movements above the table. To compare the data from different measurement sessions, scaling was performed to account for potential variations in camera and subject positioning. As the subject did not move within the frame as in the gait analysis, a single time point with a stable posture was selected as the reference, and scaling was applied using the vertical distance between the eye and shoulder levels.

- Scale Normalization

The vertical motions of the shoulder, elbow, and wrist joints were analyzed using the Y-axis. The Y-axis coordinates were scaled by dividing them by the difference between the mean Y-coordinates of the left and right inner eye landmarks and those of the left and right shoulders, which was treated as the vertical distance. All the Y-axis data were scaled using the same values.

- Upper limb Joint Height Analysis

The vertical positions of the shoulder, elbow, and wrist joints during the eating task were analyzed using the Y-axis coordinates to evaluate joint elevation. Time-series changes in the Y-axis values are visualized in graphs. In the MediaPipe coordinate system, smaller Y-axis values correspond to higher positions in the image (toward the head), whereas larger values correspond to lower positions in the image (toward the feet). Therefore, when the shoulder, elbow, or wrist was elevated, the Y-axis value decreased, and when one of them moved downward, the value increased. As this representation is opposite to the intuitive interpretation of height, further plots were generated with the Y-axis inverted.

- Peak Detection and Eating Intervals

Eating involves hand–mouth movements. Therefore, the time interval between consecutive peaks, extracted from the smoothed vertical wrist-height signal on the Y-axis, was calculated and defined as the eating interval. To identify individual eating motion cycles, peaks were detected from the signals smoothed using a moving-average filter to reduce noise. A moving-average filter with a window size of 15 frames was used. At a frame rate of 30 fps, this corresponds to approximately 0.5 s. A peak was defined as the point at which the signal transitioned from increasing to decreasing intensity. To exclude false detections, a candidate peak was accepted only if it represented the maximum value within a 1-s window. Furthermore, to ensure that the peak corresponded to significant hand-to-mouth movement, the peak amplitude was required to exceed a threshold defined as follows:

Threshold = V_min + 0.4 × (V_max − V_min),(1)

where V_max and V_min denote the maximum and minimum signal values, respectively. The 40% criterion was determined based on the assumption that a valid hand-to-mouth movement occurs when the hand reaches a height of at least 40% above the lowest hand position relative to the total signal range. This 40% threshold was determined empirically through iterative preliminary testing and informed by the clinical experience of the rehabilitation professionals involved in the study to distinguish eating gestures while minimizing false detections. Subsequently, the eating intervals were presented graphically. Various descriptive statistics, including mean, standard deviation, and minimum and maximum values, were calculated for these intervals. However, only the median was adopted as the representative metric when explaining the results to therapists to facilitate a clear and intuitive clinical interpretation.

#### 2.2.3. Table-Slide Motion Analysis

The repetitive table-slide movement used in rehabilitation was analyzed, in which a towel was moved diagonally forward to the right of the table and then returned to the starting position. Two conditions were compared: normal movement and a simulated reduction in elbow extension force accompanied by the compensatory movement of the shoulder girdle and trunk. The analysis focused on the relationship between the elbow angle of the right upper limb and the bilateral asymmetries in shoulder height and forward protraction.

- Scale Normalization

The scaling factors for each axis were determined from a single reference frame at a manually selected time point. The X-, Y-, and Z-factors were defined as the absolute differences between the right and left shoulder X-coordinates, the mean Y-coordinates of the bilateral inner eye landmarks and those of the bilateral shoulder landmarks, and the elbow and wrist Z-coordinates on the performing side. All the coordinate data were then scaled by dividing each axis by its corresponding constant factor.

- Elbow Angle

The right elbow extension angle was calculated as the three-dimensional angle between the upper arm vector (elbow–shoulder) and the forearm vector (elbow–wrist) derived from the joint coordinates.

### 2.3. Technology Acceptance Survey

#### 2.3.1. Participants

The participants included physical therapists, occupational therapists, and nurses. Participation was voluntary, and all the participants received an explanation of the study’s purpose and procedures before participation. A total of 17 participants took part in the user study.

- Study Procedure

The participants were healthcare professionals working at two hospitals in Japan. An overview of MediaPipe and the proposed motion analysis method was provided. During the session, videos of three types of gait analysis and two types of eating motion analysis were presented as examples of the proposed method. Additionally, a table-slide task was introduced as an example of an upper limb rehabilitation exercise. The introductory sessions were conducted either face-to-face or online via Zoom using PowerPoint. Subsequently, participants who wished to do so could experience the analysis by executing scripts in Google Colaboratory (Colab). All hands-on sessions were conducted via Zoom with explanations provided in real time. Example datasets and Python scripts for the MediaPipe-based analysis were shared using Google Drive. The participants accessed these resources and mounted Google Drive on Colab to execute the scripts. After the session, the participants were invited to complete a questionnaire based on the UTAUT2 framework using Google Forms. All 17 participants who took part in the study procedure completed the questionnaire (response rate: 100%). The survey was conducted between January and February 2026.

- Questionnaire Design

A questionnaire survey was conducted using the UTAUT2. The measurement indicators included the primary constructs of the UTAUT2 model: performance expectancy (PE), effort expectancy (EE), social influence (SI), facilitating conditions (FC), habit (HB), hedonic motivation (HM), price value (PV), and behavioral intention (BI). Two extended constructs were introduced to account for the specific characteristics of professionals engaged in human support services: cognitive trust (CT) and altruism (AL). In total, 10 constructs were evaluated, each measured using three items, resulting in a 30-item scale. The participants responded to these items on a five-point Likert scale ranging from 1 (strongly disagree) to 5 (strongly agree). Data on session activity types and participants’ demographic characteristics were collected to provide basic contextual information for interpreting technology acceptance. Specifically, the survey included questions regarding (1) the type of session activity, including listening to an explanation only or operating the system with a researcher-provided video; (2) the type of workplace where they had the longest experience in human support services; (3) their current profession; (4) years of experience in human support services; and (5) age. The demographic data were collected using categorical response options. The questionnaire was originally developed in Japanese and was administered to Japanese participants. The questionnaire items are presented in Table S1 (Supplementary Material).

#### 2.3.2. Statistical Analysis

Spearman’s rank correlation coefficient was used to examine associations among the questionnaire constructs and participant characteristics. To account for multiple comparisons among the 91 pairwise correlations, *p*-values were adjusted using the Benjamini–Hochberg false discovery rate (FDR) procedure. For all correlations, 95% confidence intervals were estimated using percentile bootstrap resampling with 10,000 iterations. These confidence intervals were not adjusted for multiple comparisons. Detailed results for all 91 correlations are provided in Table S2 (Supplementary Material). Differences in behavioral intention between participant subgroups were examined using the Mann–Whitney U test. These group comparisons were exploratory and addressed different participant characteristics; therefore, no adjustment for multiple comparisons was applied. Effect sizes were reported as rank-biserial correlations, with 95% confidence intervals estimated using percentile bootstrap resampling with 10,000 iterations.

## 3. Results

### 3.1. Motion Analysis

#### 3.1.1. Gait Analysis

- Time-Series Graphs of Knee and Heel Elevations

**Figure 1:**
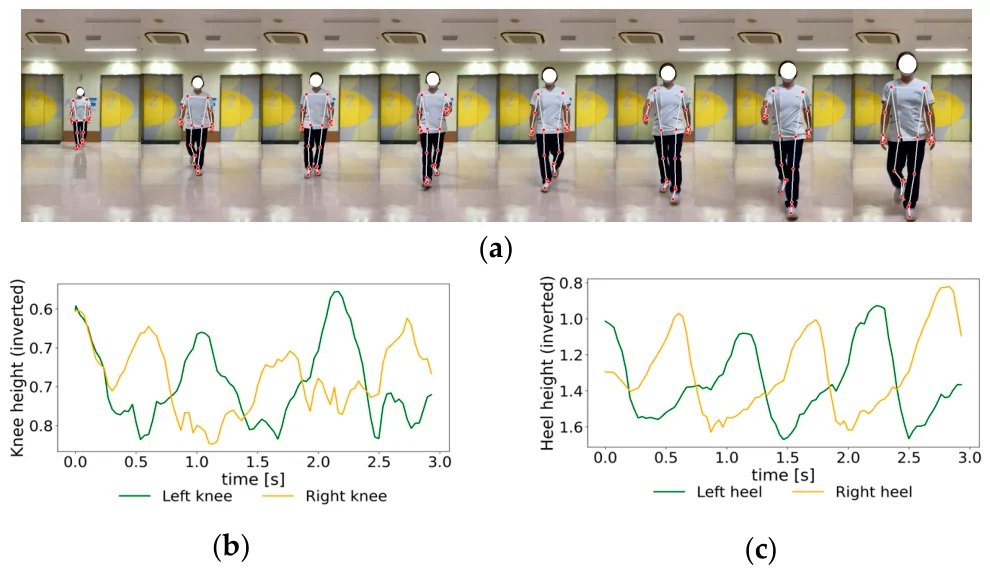
Visual representation of normal gait: (**a**) image of gait; (**b**) knee vertical displacement; (**c**) heel vertical displacement.

**Figure 2:**
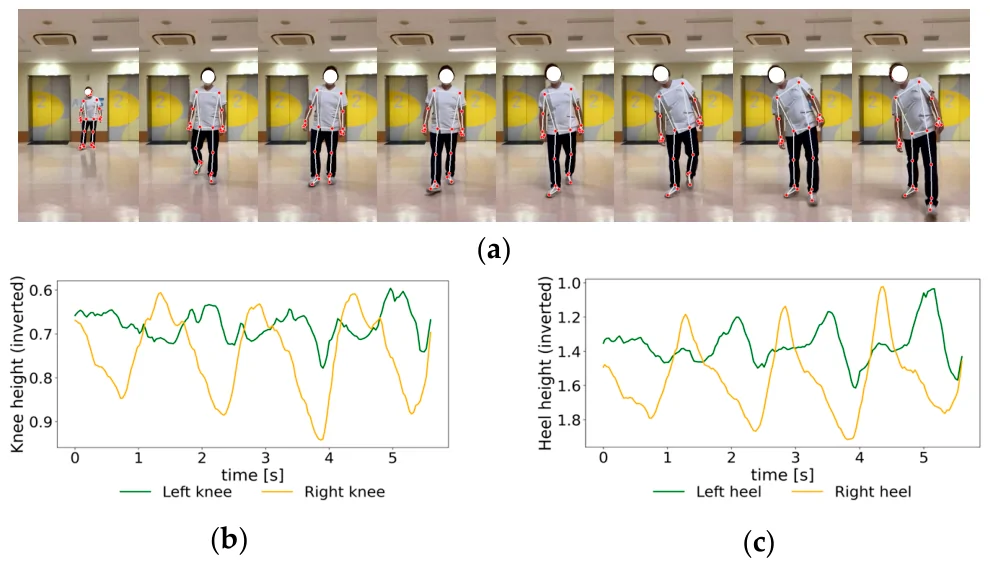
Visual representation of gait simulating right-lower-limb impairment: (**a**) image of gait; (**b**) knee vertical displacement; (**c**) heel vertical displacement.

**Figure 3:**
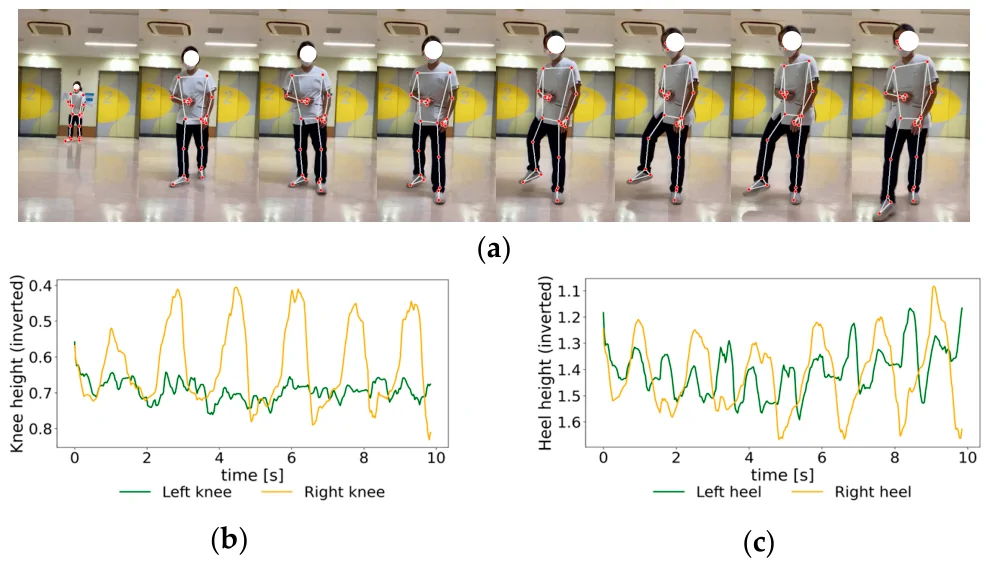
Visual representation of gait simulating right hemiparesis: (**a**) Image of gait; (**b**) Knee vertical displacement; (**c**) Heel vertical displacement.

The gait patterns under three conditions—normal gait, simulated right lower limb impairment, and simulated right hemiparesis—are shown in Figure 1, Figure 2 and Figure 3, respectively. To represent gait progression, the initial and final frames were positioned at the far left and right, respectively, with intermediate frames selected to emphasize the key characteristics. Panel (a) depicts the gait image, and panels (b) and (c) illustrate the vertical displacements of the knee and heel, respectively, as obtained by inverting the hip-to-knee and hip-to-heel distance graphs. In panels (b) and (c), the green and yellow lines represent the left and right sides, respectively. Normal gait is characterized by a high degree of left–right symmetry and regularity. In the phase corresponding to the lowest position representing foot contact, fluctuations were observed in the right knee but scarcely in the heel.

The gait simulated a right-lower-limb impairment. During the swing phase of the left lower limb, a compensatory elevation of the left pelvis occurred, accompanied by the lateral flexion and tilting of the right trunk. As the lateral flexion of the right trunk shortened the shoulder–hip distance used as the scaling baseline, the scaled distances were affected. In this analysis, the hip–knee and hip–heel distances were normalized using the shoulder–hip distance. Consequently, during the swing phase of the left lower limb, when the right foot was in contact with the ground, the shortening of the right shoulder–hip distance resulted in larger hip–knee and hip–heel distances on the right side than on the left. This result is consistent with that shown in Figure 2, in which the Y-axis is inverted. Figure 2 shows that the movements of the left lower limb were more irregular than those of the right lower limb.

The gait pattern simulating right hemiparesis is characterized by a circumduction gait with marked knee elevation. During the swing phase of the right lower limb, the right shoulder girdle was retracted, and the lateral flexion of the right trunk was observed, although it was less pronounced than that shown in Figure 2. The graph in Figure 3 indicates that the right knee was highly elevated, which is consistent with the visual observations. In addition, for the same reason as shown in Figure 2, when the trunk was laterally flexed to the right during the swing phase of the right lower limb, the hip–heel distance appeared larger.

In the inverted graph, the yellow trough is deeper than the green trough, which is consistent with the image. Furthermore, circumduction during the swing phase of the right lower limb caused instability in the supporting left knee, which appeared as a jagged waveform on the graph.

- Stride Time

The stride time is defined as the time interval between consecutive ground contacts of the same foot. The stride times were longer in the simulated right-lower-limb impaired gait and simulated right hemiparetic gait compared with those in the normal gait. The bilateral comparison showed nearly identical stride times for the normal gait. In contrast, in both the simulated right-lower-limb impaired gait and simulated right hemiparetic gait, the stride time of the right lower limb was longer than that of the left. In the simulated right-lower-limb impaired gait shown in Figure 2, the time-series gait graph indicates that the movements of the left lower limb were more irregular than those of the right. Similarly, in the simulated right hemiparetic gait shown in Figure 3, the circumduction of the right limb caused instability in the supporting left knee, which appeared as a jagged waveform in the graph. In both simulated impaired gait patterns, the irregular left stride time decreased.

#### 3.1.2. Eating Motion Analysis

- Time-Series Graphs of the Shoulder, Elbow, and Wrist Elevations

**Figure 4:**
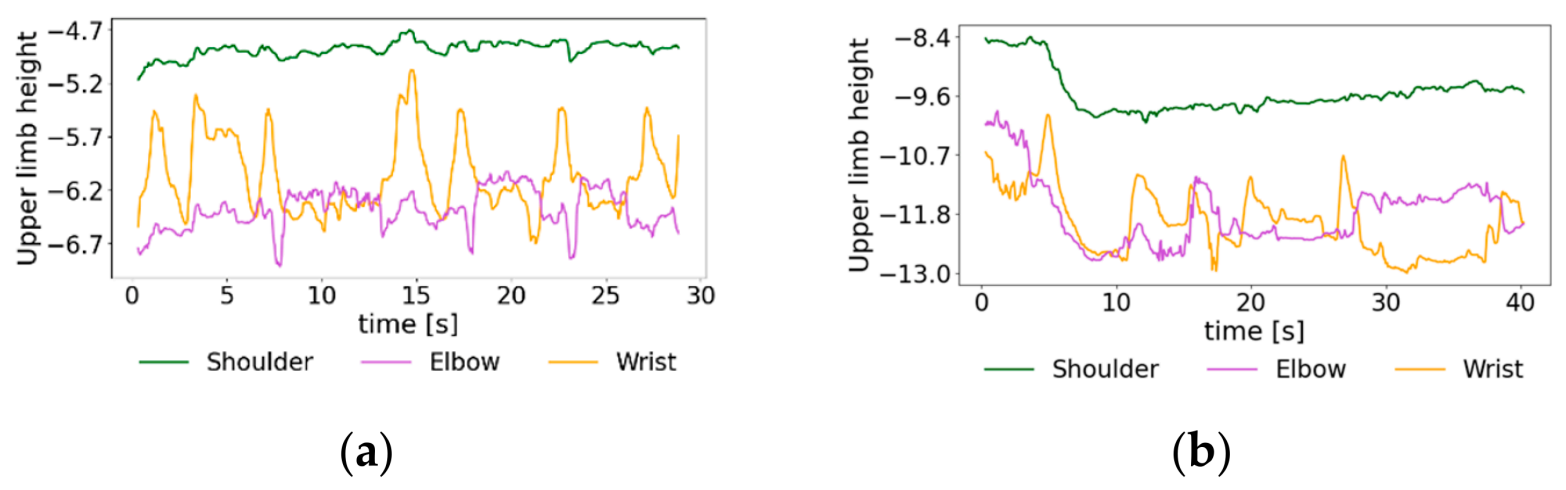
Time-series of right shoulder, elbow, and wrist heights during eating: (**a**) normal eating; (**b**) slow eating with lateral trunk flexion.

Two eating conditions were analyzed: normal eating and slow eating with the lateral flexion of the trunk, as shown in Figure 4. The corresponding time-series graphs of the right shoulder, elbow, and wrist are shown in Figure 4a,b. As MediaPipe assigns negative values to positions toward the top of the screen, the graphs in Figure 4 were inverted by multiplying the output data by −1. The graph in Figure 4b, in which the trunk is flexed laterally, appears to be more irregular than that in Figure 4a.

- Bilateral Wrist Height and Eating Pace

**Figure 5:**
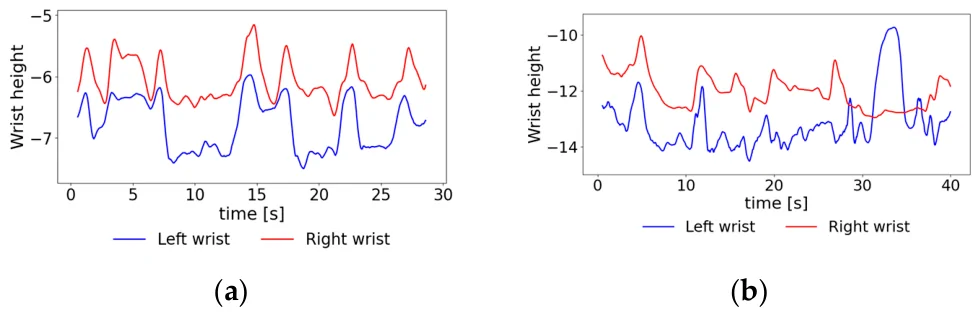
Time-series graphs of bilateral wrist height: (**a**) normal eating; (**b**) simulated eating with left lateral trunk flexion, reduced hand-movement speed, and a slower eating rhythm.

The time-series graphs of the bilateral wrist heights smoothed using a moving-average filter with a window size of 15 frames are shown in Figure 5.

**Figure 6:**
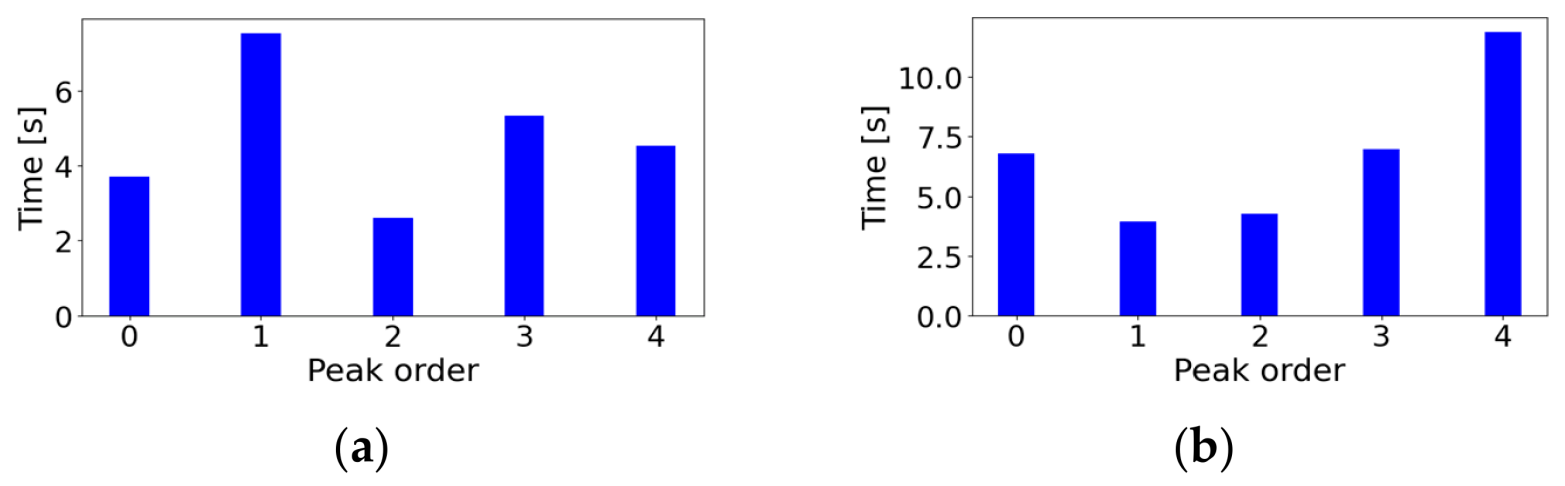
Eating intervals derived from peak detection: (**a**) normal eating; (**b**) simulated eating with left lateral trunk flexion, reduced hand-movement speed, and a slower eating rhythm.

The slow-eating condition involved drinking from a cup with the left hand. In addition, the time interval between consecutive peaks was calculated as an indicator of the eating pace. The graph is shown in Figure 6. The pace of eating is influenced by various factors, including the type of food, surrounding environment, and psychological factors. Therefore, unlike gait, it does not exhibit a constant rhythm and is not solely determined by motor function. Consequently, the required time varied between trials. However, the maximum and median values indicate that the eating condition in Figure 6b required more time than that in Figure 6a. This variability is also reflected in Figure 5b, where the elevation of the blue line for the left hand between 30 and 35 s captures the action of picking up a cup and drinking.

#### 3.1.3. Table-Slide Motion Analysis

**Figure 7:**
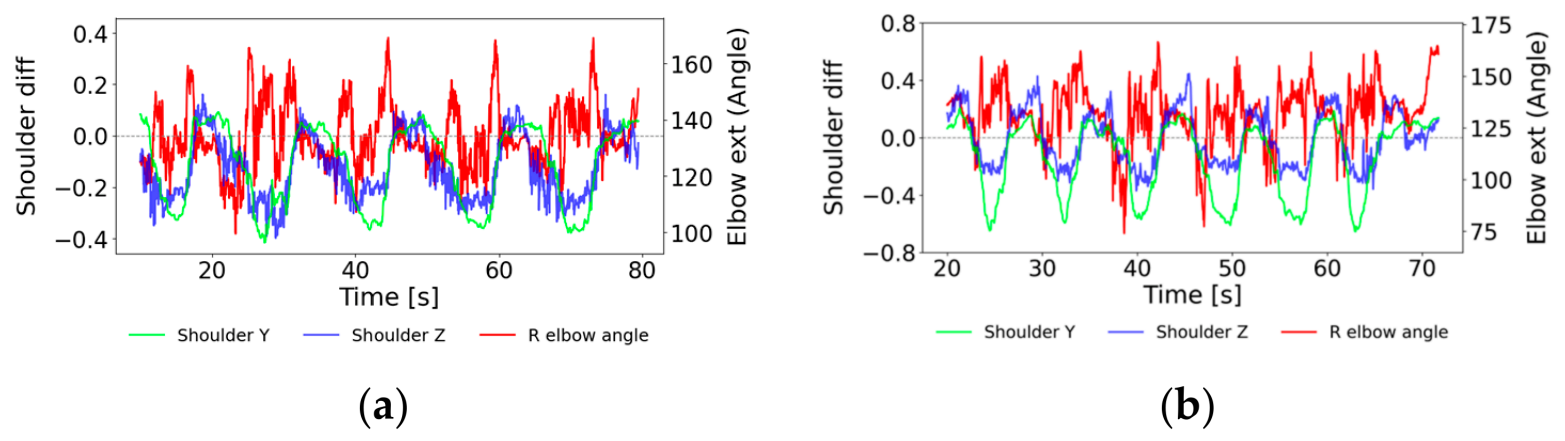
Visual representation of upper-limb movement conditions: (**a**) normal movement; (**b**) simulated movement with reduced elbow extension and compensatory shoulder movement.

One condition represented normal movement, whereas the other simulated a reduced elbow extension force accompanied by the compensatory movements of the shoulder girdle and trunk. The graphs of these conditions are shown in Figure 7.

The green line represents the bilateral difference in the Y-axis values between the shoulders, indicating the elevation of the right shoulder relative to the left. The blue line represents the difference in the Z-axis values between the shoulders, indicating the forward protraction of the right shoulder compared with the left shoulder. The red line represents the elbow extension angle, with full extension corresponding to 180°. In Figure 7, which represents the condition with pronounced shoulder girdle compensation, the blue line indicates a greater forward displacement of the shoulder. Furthermore, the trajectories demonstrated that shoulder protraction preceded elbow extension; specifically, the shoulder moved forward before the red line reached its maximum value.

### 3.2. Technology Acceptance Evaluation

#### 3.2.1. Descriptive Statistics of the UTAUT2 Constructs

Seventeen healthcare professionals participated in this survey. The participants included 12 physical therapists, 3 occupational therapists, and 2 nurses. Nine participants performed the MediaPipe analysis, whereas eight participants received the explanation only. Regarding professional experience, the largest group had 1–5 years of experience (*n* = 7), followed by 5–10 years (*n* = 5) and 11–15 years (*n* = 4). Most participants were in their 20s and 30s. The mean scores of the constructs were calculated using a five-point Likert scale. Table 1 presents the descriptive statistics of the constructs.

Among the constructs, performance expectancy had the highest mean score (M = 4.18), indicating that the participants strongly perceived the technology to be useful for improving their professional practice. Altruism also yielded a relatively high score (M = 3.98), suggesting that the participants believed that the technology would benefit patients, the healthcare sector, and society. These results indicate that healthcare professionals positively evaluate the technology in terms of both professional utility and social contributions. By contrast, effort expectancy had the lowest mean score (M = 2.71), indicating that the technology was not perceived as easy to use. An examination of individual questionnaire items further clarified this pattern: the highest overall score was observed for the facilitating conditions item “The technology could be utilized if a dedicated specialist were available” (M = 4.6), whereas the lowest score was observed for the effort expectancy item “The technology can be easily mastered” (M = 2.4). In this study, performance expectancy was assessed in terms of not only improvements in the quality of one’s own work and perceived usefulness but also its applicability to other departments. The score for applicability to other departments was relatively low (M = 3.76), whereas the scores for improvement in the quality of work (M = 4.35) and perceived usefulness (M = 4.41) were high.

Behavioral intention is important because it represents users’ intentions to use technology. While the overall mean score was 3.53, a detailed breakdown of the three-item average showed that one participant scored 5, five participants scored between 4 and 5, and one participant scored between 3 and 4, indicating that some participants were highly motivated. A comparison by profession showed that the behavioral intention scores of the physical therapists (mean = 3.78) were higher than those of the other participants (mean = 2.93; Mann–Whitney U = 52.5, *p* = 0.017, rank-biserial r = 0.75, 95% CI [0.37, 1.00]). The occupational therapists tended to have slightly lower scores (mean = 3.00, U = 6.0, *p* = 0.059, rank-biserial r = −0.71, 95% CI [−1.00, −0.19]). Behavioral intentions were also compared according to whether the participants performed MediaPipe analysis using Python during the survey. The mean behavioral intention scores were similar between the groups (experience: mean = 3.56; no experience: mean = 3.50), and the Mann–Whitney U test indicated no significant difference (U = 39.5, *p* = 0.766, rank-biserial r = 0.10, 95% CI [−0.52, 0.69]). Behavioral intention was compared based on prior experience with digital devices. The mean score was slightly higher among participants with prior experience (mean = 3.67) than among those without (mean = 3.50); however, no significant difference was observed (Mann–Whitney U = 26.0, *p* = 0.558, rank-biserial r = 0.24, 95% CI [−0.53, 0.80]).

#### 3.2.2. Internal Consistency of the Constructs

Cronbach’s alpha coefficients were calculated to assess the internal consistency. As shown in Table 2, most constructs showed acceptable to high reliability (α = 0.68–0.96). However, effort expectancy (α = 0.49), facilitating conditions (α = 0.59), and cognitive trust (α = 0.53) showed relatively lower reliability.

#### 3.2.3. Spearman Correlation Analysis

**Figure 8:**
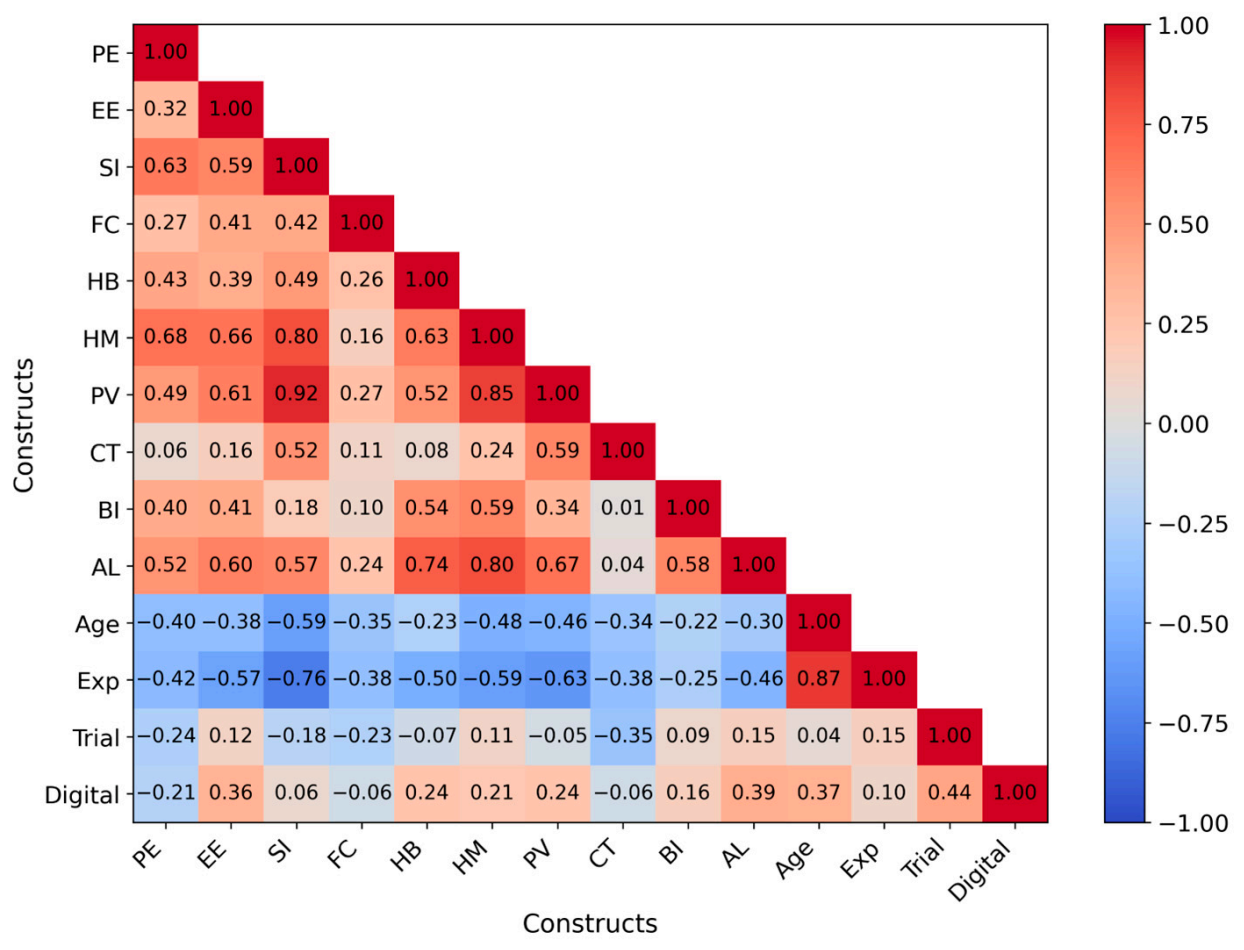
Heatmap of the Spearman correlation matrix.

The relationships among the variables are illustrated in a heatmap of the Spearman correlation matrix (Figure 8). Seven correlations exceeded 0.70. Age was strongly positively correlated with years of experience (ρ = 0.87, 95% CI [0.70, 0.98]). As this correlation was expected, it was excluded from further analysis, leaving six strong correlations for a detailed investigation.

Among these, social influence and hedonic motivation each were involved in three correlations, followed by price value and altruism (two each) and habit and years of experience (one each). Notably, years of experience showed a negative correlation with social influence. After Benjamini–Hochberg FDR correction for the 91 pairwise Spearman correlations, 13 correlations remained statistically significant. All seven strong correlations (|ρ| ≥ 0.70) remained significant (FDR-adjusted *p* < 0.01), and their bootstrap 95% confidence intervals did not include zero.

In this study, healthcare services were utilized not for the users themselves but for individuals with health challenges. Accordingly, the results are presented concerning the factors showing strong correlations, as well as altruism—a key element for such services—behavioral intention regarding future use, and performance expectancy, which showed the highest mean value. Detailed results, including unadjusted and FDR-adjusted *p*-values and 95% confidence intervals for all 91 correlations, are provided in Table S2 (Supplementary Material).

- Social influence was strongly associated with price value (ρ = 0.92, 95% CI [0.78, 0.97]) and hedonic motivation (ρ = 0.80, 95% CI [0.51, 0.94]). Moderate correlations were observed with performance expectancy (ρ = 0.63), effort expectancy (ρ = 0.59), altruism (ρ = 0.57), cognitive trust (ρ = 0.52), and habit (ρ = 0.49). In contrast, social influence exhibited a strong negative correlation with years of professional experience (ρ = −0.76, 95% CI [−0.89, −0.51]) and a moderate negative correlation with age (ρ = −0.59).
- Hedonic motivation demonstrated strong correlations with price value (ρ = 0.85, 95% CI [0.61, 0.96]), social influence (ρ = 0.80, 95% CI [0.51, 0.94]), and altruism (ρ = 0.80, 95% CI [0.54, 0.94]). Moderate correlations were also observed with performance expectancy (ρ = 0.68), effort expectancy (ρ = 0.66), habit (ρ = 0.63), and behavioral intention (ρ = 0.59).
- Price value was strongly associated with social influence (ρ = 0.92, 95% CI [0.78, 0.97]) and hedonic motivation (ρ = 0.85, 95% CI [0.61, 0.96]). Moderate correlations were observed with altruism (ρ = 0.67), effort expectancy (ρ = 0.61), cognitive trust (ρ = 0.59), habit (ρ = 0.52), and performance expectancy (ρ = 0.49).
- Altruism demonstrated strong correlations with hedonic motivation (ρ = 0.80, 95% CI [0.54, 0.94]) and habit (ρ = 0.74, 95% CI [0.45, 0.90]). Moderate correlations were observed with price value (ρ = 0.67), effort expectancy (ρ = 0.60), behavioral intention (ρ = 0.58), social influence (ρ = 0.57), and performance expectancy (ρ = 0.52).
- Habit was strongly associated with altruism (ρ = 0.74, 95% CI [0.45, 0.90]). Moderate correlations were observed with hedonic motivation (ρ = 0.63), behavioral intention (ρ = 0.54), price value (ρ = 0.52), and social influence (ρ = 0.49).
- Behavioral intention showed moderate correlations with hedonic motivation (ρ = 0.59), altruism (ρ = 0.58), and habit (ρ = 0.54).
- Performance expectancy showed no strong correlation with any variables. However, moderate correlations were observed with hedonic motivation (ρ = 0.68) and social influence (ρ = 0.63). In addition, moderate correlations were observed with habit (ρ = 0.43), price value (ρ = 0.49), behavioral intention (ρ = 0.40), and altruism (ρ = 0.52). Performance expectancy also showed negative correlations with age (ρ = −0.40) and years of experience (ρ = −0.42).

#### 3.2.4. Free-Response Comment Results

The survey included two types of free-response questions. The results are summarized below.

- Qualitative Feedback on System Usability

All free-response comments were provided by the physical therapists regarding feedback on the technology analysis using MediaPipe. The responses were organized into the following four primary categories (hereafter, PT1–PT4 denote the different physical therapists and NS1 denotes a nurse):

Clinical Utility and Applicability: The participants expressed high expectations regarding the clinical application of MediaPipe and indicated that they would consider using the system in clinical practice if distance measurements became available. “The ability to estimate the sagittal plane motion from frontal videos may reduce the time required for gait analysis. Additionally, it may enable the measurement of distances that cannot be determined from videos alone, thereby facilitating its use in clinical settings.”

(PT1)

Usefulness as a Visual Feedback Tool: Visual information, such as landmark trajectories and graphical representations, is considered useful for enhancing patient understanding and supporting clinical explanations. “The visualization of landmark movements through graphs makes it easier for patients to understand them, which I think is particularly beneficial.”

(PT2)

Expectations of Increased Utility with Familiarization: The participants suggested that, although some familiarization may be required, the system could become highly beneficial in clinical practice once users become accustomed to MediaPipe. “Once I become accustomed to MediaPipe, I believe it could be very useful clinically.”

(PT3)

Concerns Regarding Analytical Methods and Technical Limitations: The participants raised concerns regarding analytical procedures and computational logic. Specifically, questions were raised about depth estimation, the accuracy of three-dimensional motion analysis, responsiveness to variations in movement speed, and limitations in evaluating trunk motion using distance-based measures. In addition, interpreting coordinate data requires a certain level of specialized knowledge and proficiency. “I feel that it is difficult to interpret the data without a clear understanding of how to handle coordinate data and what kind of information should be extracted.” (PT3) “How is depth determined from planar video? How are the differences in movement speeds handled? If the distances between two points were used, would it be difficult to evaluate trunk movements?”

(PT4)

- Free-response Feedback on the Medical Reimbursement System

Furthermore, participants who provided a positive response (ratings of 4 or 5 on a five-point scale) to the item “I would actively use this technology depending on the reimbursement system” were asked to provide free-text responses regarding the reimbursement systems required for its implementation. Responses were obtained from both physical therapists and nurses, and the following institutional considerations were identified:

Evaluation of Motion Analysis and Technical Accuracy: The participants expressed expectations for the introduction of reimbursement for motion analysis, provided that accuracy comparable to conventional three-dimensional motion analysis could be ensured. In addition, expectations were expressed regarding the official recognition of AI-based motion analysis within the reimbursement system. “I expect that if quality can be ensured at a level comparable to three-dimensional motion analysis, AI-based motion analysis may eventually be accepted within the reimbursement system.”

(PT3)

Evaluation Based on Clinical Effectiveness: A nurse suggested that demonstrating tangible clinical benefits, such as improved rehabilitation effectiveness and reduced rehabilitation duration, would be necessary for reimbursement approval. Improvements in the orthotic selection accuracy have also been suggested as potential factors. “If it can be demonstrated that this tool improves rehabilitation effectiveness and shortens the rehabilitation period, it may be recognized for reimbursement. It may also be acceptable if it improves the accuracy of the orthotic selection.”

(NS1)

## 4. Discussion

Although MediaPipe outputs 99 coordinates, this study utilized only a limited subset: for gait analysis, eight variables consisting of the bilateral Y-coordinates of the shoulder, hip, knee, and ankle; for eating, eight variables consisting of the bilateral Y-coordinates of the eye, shoulder, elbow, and hand; and for the table-slide task, 12 variables comprising the X, Y, and Z coordinates of the shoulder, elbow, and wrist. Despite this limited dataset, the questionnaire results yielded a very high score for the item “The technology could be utilized if a dedicated specialist were available” (mean = 4.6 on a five-point scale).

### 4.1. Motion Analysis Using MediaPipe

An analysis of the three gait patterns revealed the characteristics of each condition. The normal gait demonstrated high symmetry and regularity. In the simulated right-lower-limb impairment condition, characterized by the compensatory elevation of the left pelvis during the left swing phase accompanied by rightward lateral trunk bending, the left limb exhibited greater irregularity than the right limb. The simulated right hemiparetic gait showed a circumduction pattern with pronounced right knee elevation, which was reflected by an increased right knee displacement. Additionally, the circumductive movement of the right limb during swinging appeared to induce instability in the supporting left knee, which can be observed as jagged waveforms in the corresponding graphs.

In gait analysis, scaling was performed by dividing the hip–knee and hip–heel vertical distances by the shoulder–hip distance at each timestamp. In both simulated right-lower-limb impaired and hemiparetic gaits, lateral trunk bending toward the right side shortened the shoulder–hip distance used as the scaling reference. Consequently, the calculated hip–knee and hip–heel distances appeared larger than their actual values. However, these apparent left–right differences may reflect the combined effects of lower-limb movement and shortening of the shoulder–hip scaling baseline associated with right trunk lateral flexion and should therefore not be interpreted as representing lower-limb kinematics alone. As the positions of the left and right feet during ground contact are at the same height on the floor, this consistency should be considered in future calculations. Even during a normal gait, fluctuations were observed in the right knee at the moment of foot contact, whereas instability was rarely observed in the heel. A visual confirmation of the video by a physical therapist did not reveal the actual joint instability, suggesting that these fluctuations may originate from the tendency of MediaPipe to produce less stable estimates for the knee than for the heel. However, as similar fluctuations were not observed in the left knee, the reliability of the Y-coordinate extraction for the knee remains a challenge for future work. In the stride-time calculations, irregular patterns were observed during the stance phase of the left lower limb, which acted as support for the impaired right limb. These findings underscore the importance of capturing the behavior of both impaired and contralateral supporting limbs.

For the eating task, the time-series Y-coordinate positions of the shoulders, elbows, and hands were analyzed. This enabled the assessment of coordination among these joints as well as the pacing of food intake, allowing differences between the two types of eating movements to be observed. In eating activities, not only the ability to self-feed, but also the appropriateness of pacing is important. In individuals with cognitive impairment, pacing disturbances, such as rapid eating or food-stuffing, can occur and may lead to dietary restrictions [58,59]. Conversely, excessively slow eating may result in an insufficient nutritional intake. Therefore, pacing is an important indicator in the assessment of eating behavior. In the table-slide task, when compensatory elbow flexion by the shoulder girdle was present, the anterior displacement of the shoulder preceded elbow extension. This finding showed differences between the two movement patterns. Compensatory shoulder movements were also captured in the table-slide task, reflecting compensation for reduced elbow extension strength. These findings suggest that, even with a limited set of variables, the key features of simulated gait, eating, and table-slide movements can be effectively captured, providing preliminary support for the feasibility of the proposed method. Unlike recent machine learning-assisted approaches using advanced dedicated sensors [74], the present approach uses only a single RGB camera, prioritizing accessibility and ease of implementation in clinical settings. Furthermore, when recording the activities of daily living, a careful consideration of privacy is required, particularly for eating activities, which are meaningful behaviors associated with enjoyment and cultural contexts.

### 4.2. Technology Acceptance and Feasibility

In the questionnaire results, the item stating that “The technology could be utilized if a dedicated specialist were available” yielded the highest score (mean = 4.6). In contrast, the item “The technology can be easily mastered” showed the lowest score (mean = 2.4). These results suggest that, while healthcare professionals recognize the potential usefulness of the technology, they perceive difficulties in operating it without specialized support.

Based on the Cronbach’s alpha results, most constructs demonstrated acceptable to high reliability, indicating that the questionnaire achieved satisfactory internal consistency overall. However, effort expectancy, facilitating conditions, and cognitive trust exhibited lower internal consistency than the other constructs. A possible explanation for the lower internal consistency of effort expectancy is that the participants had not yet used the technology in their routine work. Therefore, their responses were based on demonstrations and expectations rather than actual hands-on experience, which may have contributed to the increased variability in their evaluations. In addition, facilitating conditions exhibited relatively low internal consistency. This may be attributed to the fact that the items capture multiple dimensions of organizational support, including the institutional environment, available resources, and the presence of specialized personnel. As this technology has not yet been implemented in routine work, the participants may have assessed these conditions based on assumptions about their workplace environments, thereby contributing to the variability in responses. Cognitive trust may also reflect diverse dimensions, including data reliability, privacy protection, and system performance, which may lead to response variability. Note that each construct in this study consisted of only three questionnaire items, and the sample size was relatively small (*n* = 17), which may have influenced the stability of the reliability estimates. Considering the exploratory nature of this study, the findings should be interpreted with caution. In future studies with larger sample sizes, we plan to include Effort Expectancy in analyses examining the relationship between hands-on experience and technology acceptance.

Based on the mean values, performance expectancy had the highest score, followed by altruism. As performance expectancy and altruism are considered key factors in the acceptance of healthcare technologies, the high scores suggest that the implementation of this technology is likely to be positively perceived. Based on Spearman’s correlation analysis, performance expectancy was observed to be relatively strongly associated with hedonic motivation and social influence. In addition, altruism showed a moderate to strong correlation with several constructs. In particular, altruism was strongly correlated with hedonic motivation and habit and moderately correlated with price value and behavioral intention. These results suggest that the more healthcare professionals are interested in and positively perceive technology, the more they tend to perceive greater patient-related benefits and stronger expectations for its adoption in clinical practice. Furthermore, technologies that are more socially recognized may enhance expectations regarding their application in professional practice. Altruism is strongly correlated with hedonic motivation and habit. These results suggest that individuals who perceive technology as beneficial to others may also experience greater enjoyment in using it and may be more likely to develop habitual engagement with technology. In healthcare settings, where technologies are often adopted to improve patient outcomes rather than for personal benefit, altruistic attitudes play an important role in shaping the positive perceptions of technology use. Additionally, moderate correlations among price value, effort expectancy, behavioral intention, social influence, and performance expectancy indicate that altruistic motivation may be associated with multiple dimensions of technology perception, including perceived usefulness and usability. Collectively, these findings suggest that an altruistic perspective contributes to a positive attitude toward the implementation of healthcare technologies.

A notable finding from the Spearman correlation analysis was that years of experience were negatively correlated with all the technology acceptance constructs, including performance expectancy, effort expectancy, social influence, facilitating conditions, habit, hedonic motivation, price value, cognitive trust, behavioral intention, and altruism. This association suggests that healthcare professionals with more years of experience may be more critical or cautious in their evaluation of technology across all dimensions of acceptance. Notably, the negative correlation with social influence suggests that more experienced professionals may be less influenced by social expectations when evaluating technology. This association may suggest that more experienced healthcare professionals rely more on their own clinical experience and professional knowledge, rather than on external evaluations or peer opinions, when deciding whether to adopt a new technology.

Therefore, while social recognition and interprofessional support play crucial roles in technology acceptance, clearly demonstrating evidence and clinical utility remains essential for promoting technology implementation in the medical field. In the present study, years of experience showed a pattern similar to that of age. A previous study has also reported that older age is associated with a lower acceptance of digital health technologies, whereas younger physiotherapists use sensors, wearables, and web-based applications more frequently [75]. Although the underlying mechanisms could not be identified in this study, these findings may suggest that, in addition to accumulated clinical experience, age-related differences in familiarity with digital technologies could play a role. Differences associated with the so-called “digital native” generations may also contribute to this trend.

Hedonic motivation showed strong correlations with price value, social influence, and altruism, indicating that it was not merely associated with entertainment, but was intrinsically linked to social recognition, value perception, and altruistic significance. In particular, a very strong correlation was observed between hedonic motivation and altruism (ρ = 0.80). These findings suggest that perceptions of social support, value, and benefit to others may be associated with greater enjoyment and more positive emotional responses toward technology use. Therefore, in the healthcare context, technologies recognized as socially meaningful and beneficial for patients may be more likely to induce positive perceptions and acceptance among healthcare professionals, which may be considered a characteristic feature of healthcare. Behavioral intention is a primary determinant of technology acceptance and represents a user’s intent to adopt a technology. While Spearman’s correlation analysis did not reveal any strong correlations, behavioral intention showed moderate correlations with hedonic motivation, altruism, and habit. This suggests that behavioral intention may be primarily influenced by intrinsic factors. Although the overall mean score was 3.53, one participant achieved a maximum score of 5 across all items, and five participants scored between 4 and 5, indicating that some participants were highly motivated. Furthermore, the physical therapists had a higher mean behavioral intention score (3.78) than the other participants. This may be attributed to their strong interest in motion analysis, a professional culture centered on gait analysis, and extensive biomechanics education. Spearman’s correlation analysis revealed that social influence, price value, and hedonic motivation are strongly correlated. These findings suggest that socially recognized technologies are more likely to be perceived as valuable and may elicit more positive emotional responses toward their use.

Qualitative responses suggested that time efficiency in analysis and visualization was well received by the physical therapists to meet their clinical needs. The identified challenges included the need for transparency in the calculation logic and user training. Furthermore, for real-world implementation, demonstrating not only accuracy but also clinical outcomes, such as reduced intervention time, may be key to integration into reimbursement systems. Based on these findings, it is considered that, in addition to technical performance, clinical usefulness and appropriate operational implementation are important for the acceptance and dissemination of this technology. In addition, the findings of the questionnaire survey and correlation analyses should be interpreted with caution because of the exploratory nature of this study and the relatively small sample size. Although several moderate-to-strong correlations were observed, these findings are preliminary and should not be taken as definitive evidence of relationships among the constructs. The observed correlations indicate associations among the constructs and should not be interpreted as evidence of causal relationships. Future studies with larger sample sizes are required to confirm these findings and improve their statistical robustness. The selected movement parameters were intended to demonstrate the feasibility of the proposed approach and should not be regarded as a comprehensive set of clinical assessment parameters. Future studies should select and validate parameters according to the target disease and functional impairment. A limitation of this study is that the proposed method was evaluated only under simulated impairment conditions, which may not fully reflect the movements observed in actual patients. Future studies should validate the proposed method in clinical populations and further establish its technical validity.

### 4.3. Implications for Clinical Implementation and Future Directions

However, detailed assessments are not always required. Simplicity is important for continuous monitoring, and screening is initially employed to obtain a broad overview. The time required for operation and comprehension is a critical factor in technology acceptance. For daily monitoring or screening, the process should be feasible within a short timeframe. Based on the post-analysis discussion between the physical and occupational therapists involved in the motion analysis, approximately one minute was considered a practical target for reviewing the summarized results. However, the target duration for data review and clinical judgment was not directly derived from the results of this study or quantitatively evaluated; therefore, it remains a preliminary estimate and should be validated in future clinical studies. The importance of such brief assessments aligns with previous studies emphasizing the need for concise data presentation. Conversely, it is equally important to enable more precise analysis when a detailed evaluation is necessary. Prior studies have noted that a system that provides both concisely summarized data and the option to review the details is ideal [21]. This suggests the need for a dual-layer information structure consisting of both simplified and detailed data, reflecting a hybrid approach. The necessity for detailed data was also reflected in the open-ended responses in the present study. These aspects indicate the need to distinguish between simple automated evaluations using devices and detailed evaluations based on in-depth data analyses. Currently, a detailed analysis may require clinical interpretation by specialists, rather than relying solely on the device. While the average score for behavioral intention was 3.53, some participants exhibited high intentions across all items, suggesting that a subset of users was motivated to perform detailed analyses based on device-generated data. This implies that a specific group of healthcare professionals could fulfill the demand for a detailed evaluation by taking on the role of specialized analysts. Achieving this division of roles requires targeted education for healthcare professionals. In addition to general digital literacy training, it is important to provide advanced education to interested healthcare professionals. Furthermore, beyond human education, the development of devices with intuitive user interfaces and refined analytical methods may help reduce the cognitive load on specialists. Successful implementation of the proposed system in clinical practice will require not only technical improvements but also organizational support, including staff training and workflow integration. Future clinical implementation of video-based motion analysis also requires consideration of regulatory, privacy, and cost-related issues. Video recordings may contain identifiable personal information; therefore, their acquisition, storage, processing, and use must comply with applicable ethical and legal requirements, including privacy and image-rights regulations, which may vary across countries and institutions. Although the proposed approach does not require dedicated motion-capture equipment or wearable sensors, the costs associated with computing resources, data management, and operational infrastructure should also be considered. These aspects should be addressed in future implementation studies. With the widespread adoption of smartphones, activity recognition technology in daily life continues to advance [1,3]. The essence of activity assessment lies in measurements within real-world environments, rather than being limited to clinical or institutional settings. The advancement of digital activity assessment will depend on the integration of technical improvements, such as reliability, validity, ease of use, and supportive organizational and social structures, including specialized education.

## 5. Conclusions

This study demonstrated the feasibility of using MediaPipe to analyze essential movements in gait, eating, and table-sliding tasks under simulated conditions, even with a limited set of variables. The findings suggest the feasibility of capturing kinematic characteristics, including compensatory movements in gait impairment and movement patterns in activities of daily living, using the proposed approach. Regarding technology acceptance, although positive evaluations were obtained for the clinical utility of the system, challenges related to operability became evident, suggesting the need for specialized technical support. For successful implementation in clinical settings, it is crucial to provide rapid and intuitively understandable data presentation while simultaneously establishing a framework that allows detailed evaluation when necessary. The finding that some participants exhibited high behavioral intention suggests that a group of healthcare professionals may perform advanced analyses based on device-generated data. Future studies should focus on validating the proposed method in clinical populations, improving data reliability and validity, enhancing user interfaces, establishing educational frameworks for healthcare professionals, and developing an approach that combines simple device-based assessments with expert-driven detailed analysis. By integrating these technical and social factors, digital motion analysis is expected to contribute to more effective and objective activity assessment in real-world clinical settings.

## Figures and Tables

**Table 1:** Means and standard deviations of UTAUT2 constructs.

Construct	Mean	SD
Performance Expectancy (PE)	4.18	0.55
Effort Expectancy (EE)	2.71	0.48
Social Influence (SI)	3.78	0.86
Facilitating Conditions (FC)	3.90	0.60
Habit (HB)	3.25	0.84
Hedonic Motivation (HM)	3.84	0.68
Price Value (PV)	3.67	0.84
Cognitive Trust (CT)	3.53	0.66
Behavioral Intention (BI)	3.53	0.67
Altruism (AL)	3.98	0.92

**Table 2:** Cronbach’s alpha values for the constructs.

Construct	PE	EE	SI	FC	HB	HM	PV	CT	BI	AL
Cronbach alpha	0.79	0.49	0.82	0.59	0.87	0.68	0.87	0.53	0.94	0.95

## Data Availability

The datasets presented in this study are not publicly available because informed consent obtained from the participants was limited to the use of data for this study and did not include permission for public data sharing. Requests to access the datasets should be directed to the corresponding author.

## Supplementary Materials

The following supporting information can be downloaded at https://www.mdpi.com/article/10.3390/s26175681/s1. Table S1: Questionnaire items based on UTAUT2; Table S2: Spearman’s rank correlations with 95% confidence intervals and FDR-adjusted *p*-values.
