# Supplementary material for: MediaPipe-Based Activity Analysis by Healthcare Professionals: Method Development and Technology Acceptance—A Pilot Study
Source: Sensors (Basel). 2026 Sep 7;26(17):5681. doi: 10.3390/s26175681 (PMC13568338; doi:10.3390/s26175681)
Supplement: Supplementary file 1 [file sensors-26-05681-s001.zip › Table S2. Spearman Correlations.pdf]

Table S2. Spearman's rank correlations with 95% confidence intervals and FDR-adjusted p-values

Var. 1	Var. 2	ϱ	95% CI	p	FDR p	Var. 1	Var. 2	ϱ	95% CI	p	FDR p	Var. 1	Var. 2	ϱ	95% CI	p	FDR p
SI	PV	0.92	[0.78, 0.97]	<0.001	<0.001	PV	Age	-0.46	[-0.84, 0.11]	0.061	0.174	PE	Trial	-0.24	[-0.67, 0.27]	0.360	0.521
Age	Exp	0.87	[0.70, 0.98]	<0.001	<0.001	AL	Exp	-0.46	[-0.82, 0.04]	0.063	0.174	FC	Trial	-0.23	[-0.69, 0.26]	0.367	0.521
HM	PV	0.85	[0.61, 0.96]	<0.001	<0.001	Trial	Digital	0.44	[0.21, 0.70]	0.080	0.214	HB	Age	-0.23	[-0.69, 0.32]	0.373	0.522
SI	HM	0.80	[0.51, 0.94]	<0.001	0.002	PE	HB	0.43	[-0.07, 0.77]	0.087	0.225	BI	Age	-0.22	[-0.66, 0.31]	0.393	0.542
HM	AL	0.80	[0.54, 0.94]	<0.001	0.002	SI	FC	0.42	[-0.13, 0.80]	0.094	0.231	PE	Digital	-0.21	[-0.54, 0.13]	0.414	0.562
SI	Exp	-0.76	[-0.89, -0.51]	<0.001	0.006	PE	Exp	-0.42	[-0.75, 0.03]	0.094	0.231	HM	Digital	0.21	[-0.18, 0.57]	0.425	0.569
HB	AL	0.74	[0.45, 0.90]	<0.001	0.008	EE	BI	0.41	[-0.11, 0.76]	0.101	0.243	SI	BI	0.18	[-0.39, 0.66]	0.480	0.627
PE	HM	0.68	[0.30, 0.89]	0.003	0.031	EE	FC	0.41	[-0.11, 0.81]	0.105	0.244	SI	Trial	-0.18	[-0.66, 0.33]	0.482	0.627
PV	AL	0.67	[0.25, 0.93]	0.003	0.033	PE	BI	0.40	[-0.16, 0.83]	0.110	0.249	BI	Digital	0.16	[-0.35, 0.60]	0.533	0.680
EE	HM	0.66	[0.24, 0.87]	0.004	0.037	PE	Age	-0.40	[-0.79, 0.09]	0.116	0.253	EE	CT	0.16	[-0.36, 0.59]	0.545	0.680
PE	SI	0.63	[0.18, 0.91]	0.006	0.049	EE	HB	0.39	[-0.11, 0.78]	0.117	0.253	FC	HM	0.16	[-0.45, 0.66]	0.545	0.680
HB	HM	0.63	[0.12, 0.91]	0.007	0.049	AL	Digital	0.39	[0.05, 0.68]	0.122	0.258	Exp	Trial	0.15	[-0.39, 0.70]	0.559	0.687
PV	Exp	-0.63	[-0.85, -0.23]	0.007	0.049	FC	Exp	-0.38	[-0.76, 0.09]	0.131	0.269	AL	Trial	0.15	[-0.35, 0.61]	0.568	0.690
EE	PV	0.61	[0.16, 0.86]	0.009	0.058	CT	Exp	-0.38	[-0.76, 0.11]	0.133	0.269	EE	Trial	0.12	[-0.39, 0.59]	0.636	0.762
EE	AL	0.60	[0.27, 0.80]	0.011	0.060	EE	Age	-0.38	[-0.77, 0.17]	0.136	0.269	FC	CT	0.11	[-0.39, 0.62]	0.668	0.788
EE	SI	0.59	[0.18, 0.85]	0.012	0.060	Age	Digital	0.37	[0.06, 0.77]	0.146	0.284	HM	Trial	0.11	[-0.40, 0.58]	0.676	0.788
SI	Age	-0.59	[-0.87, -0.15]	0.013	0.060	EE	Digital	0.36	[-0.08, 0.69]	0.161	0.304	Exp	Digital	0.10	[-0.25, 0.50]	0.703	0.810
HM	Exp	-0.59	[-0.86, -0.16]	0.013	0.060	FC	Age	-0.35	[-0.70, 0.10]	0.171	0.316	FC	BI	0.10	[-0.47, 0.59]	0.717	0.815
HM	BI	0.59	[0.09, 0.86]	0.013	0.060	CT	Trial	-0.35	[-0.79, 0.18]	0.173	0.316	BI	Trial	0.09	[-0.42, 0.57]	0.740	0.832
PV	CT	0.59	[0.09, 0.87]	0.014	0.060	CT	Age	-0.34	[-0.72, 0.21]	0.182	0.325	HB	CT	0.08	[-0.51, 0.65]	0.764	0.848
BI	AL	0.58	[0.09, 0.93]	0.014	0.060	PV	BI	0.34	[-0.23, 0.79]	0.187	0.328	HB	Trial	-0.07	[-0.60, 0.44]	0.780	0.849
SI	AL	0.57	[0.09, 0.90]	0.016	0.068	PE	EE	0.32	[-0.14, 0.68]	0.215	0.369	CT	Digital	-0.06	[-0.58, 0.47]	0.805	0.849
EE	Exp	-0.57	[-0.84, -0.14]	0.017	0.068	AL	Age	-0.30	[-0.73, 0.23]	0.247	0.416	FC	Digital	-0.06	[-0.47, 0.36]	0.806	0.849
HB	BI	0.54	[0.08, 0.84]	0.025	0.095	PE	FC	0.27	[-0.32, 0.73]	0.291	0.481	SI	Digital	0.06	[-0.35, 0.46]	0.808	0.849
SI	CT	0.52	[0.04, 0.85]	0.030	0.110	FC	PV	0.27	[-0.27, 0.69]	0.296	0.481	PE	CT	0.06	[-0.44, 0.54]	0.811	0.849
PE	AL	0.52	[-0.04, 0.88]	0.032	0.110	FC	HB	0.26	[-0.33, 0.72]	0.316	0.504	PV	Trial	-0.05	[-0.57, 0.46]	0.854	0.883
HB	PV	0.52	[-0.03, 0.88]	0.034	0.114	BI	Exp	-0.25	[-0.70, 0.31]	0.337	0.521	Age	Trial	0.04	[-0.47, 0.59]	0.878	0.896
HB	Exp	-0.50	[-0.84, -0.02]	0.039	0.127	HM	CT	0.24	[-0.32, 0.70]	0.347	0.521	CT	AL	0.04	[-0.54, 0.59]	0.886	0.896
PE	PV	0.49	[-0.02, 0.84]	0.046	0.141	HB	Digital	0.24	[-0.15, 0.59]	0.353	0.521	CT	BI	0.01	[-0.61, 0.57]	0.966	0.966
SI	HB	0.49	[-0.03, 0.86]	0.046	0.141	PV	Digital	0.24	[-0.31, 0.66]	0.358	0.521						
HM	Age	-0.48	[-0.84, 0.04]	0.051	0.150	FC	AL	0.24	[-0.31, 0.66]	0.359	0.521						

Note: n = 17 for all correlations. FDR p-values were calculated using the Benjamini–Hochberg procedure. 95% CIs were estimated using percentile bootstrap resampling with 10,000 iterations.

Abbreviations: PE, Performance Expectancy; EE, Effort Expectancy; SI, Social Influence; FC, Facilitating Conditions; HB, Habit; HM, Hedonic Motivation; PV, Price Value; AL, Altruism; CT, Cognitive Trust; BI, Behavioral Intention; Age, age category; Exp, years of professional experience; Trial, type of session activity; Digital, prior experience with digital devices.
