# Supplementary material for: MediaPipe-Based Activity Analysis by Healthcare Professionals: Method Development and Technology Acceptance—A Pilot Study
Source: Sensors (Basel). 2026 Sep 7;26(17):5681. doi: 10.3390/s26175681 (PMC13568338; doi:10.3390/s26175681)
Supplement: Supplementary file 1 [file sensors-26-05681-s001.zip › Table S1. Questionnaire items based on UTAUT2.pdf]

Table S1. Questionnaire items based on UTAUT2

Section	Item Code	Item	Response Options
Session	D0	Please indicate the activity performed today.	1=Explanation only; 2=Operated MediaPipe using researcher-provided videos; 3=Operated MediaPipe using videos prepared by the participant
Demographics	D1	Type of workplace where you have had the longest experience in human support services	1=Hospital/Clinic; 2=Home-visit (medical); 3=Home-visit (long-term care); 4=Residential facility; 5=Day-care facility; 6=Combined residential/day-care facility; 7=Other
Demographics	D2	Your profession (multiple answers allowed)	Occupational therapist; Physical therapist; Speech therapist; Nurse; Public health nurse; Care worker; Care manager; Social worker; Other
Demographics	D3	Years of experience in human support services (total years, with part-time work converted to full-time equivalent based on a 40-hour workweek).	1=<1 year; 2=1–<5 years; 3=5–<10 years; 4=10–<15 years; 5=15–<20 years; 6=20–<25 years; 7=25–<30 years; 8=30–<35 years; 9=35–<40 years; 10=≥40 years
Demographics	D4	Age group	1=≤19; 2=20–29; 3=30–39; 4=40–49; 5=50–59; 6=60–69; 7=≥ 70
PE	PE1	Using this technology improves the quality of my work.	5-point Likert scale
PE	PE2	This technology is useful for my work.	5-point Likert scale
PE	PE3	This technology is useful not only for my department but also for other departments in the organization.	5-point Likert scale
EE	EE1	This technology is easy to use.	5-point Likert scale
EE	EE2	It is easy to learn how to operate this technology.	5-point Likert scale
EE	EE3	I can easily become proficient in using this technology.	5-point Likert scale
SI	SI1	This technology is expected by the people I serve.	5-point Likert scale
SI	SI2	My workplace would encourage the use of this technology.	5-point Likert scale
SI	SI3	Society, government agencies, and professional organizations would encourage the use of this technology.	5-point Likert scale
FC	FC1	This technology could be utilized under the current medical reimbursement system, provided adequate conditions are met.	5-point Likert scale
FC	FC2	I would actively use this technology depending on reimbursement conditions.	5-point Likert scale

FC	FC_free	[Only for respondents who selected 4 or 5 in FC2] Please freely describe the reimbursement system that you consider necessary to utilize this technology.	Free-text response
FC	FC3	This technology can be utilized if a dedicated specialist is newly assigned.	5-point Likert scale
HB	HB1	This technology will become widely used in society in the future.	5-point Likert scale
HB	HB2	This technology will become routinely utilized as part of my daily work.	5-point Likert scale
HB	HB3	This technology will become routinely utilized in the daily work of other departments within the workplace.	5-point Likert scale
HM	HM1	Using this technology makes me feel positive about my work.	5-point Likert scale
HM	HM2	Using this technology is interesting.	5-point Likert scale
HM	HM3	Utilizing this technology is enjoyable, as it helps reduce workload.	5-point Likert scale
PV	PV1	This technology is worth the cost of implementation.	5-point Likert scale
PV	PV2	Using this technology is cost-effective.	5-point Likert scale
PV	PV3	This technology provides value beyond its cost (including free use).	5-point Likert scale
CT	CT1	The data obtained using this technology are reliable for professional use.	5-point Likert scale
CT	CT2	The data obtained using this technology are adequately protected in terms of privacy.	5-point Likert scale
CT	CT3	This technology operates as expected.	5-point Likert scale
BI	BI1	I intend to use this technology in the future.	5-point Likert scale
BI	BI2	I intend to work toward being able to use this technology.	5-point Likert scale
BI	BI3	I intend to work toward further utilizing the data obtained using this technology.	5-point Likert scale
AL	AL1	This technology is beneficial for service users.	5-point Likert scale
AL	AL2	This technology is beneficial for healthcare and welfare fields.	5-point Likert scale
AL	AL3	This technology benefits society as a whole.	5-point Likert scale
Additional	D5	Current workplace type (select one)	Same as D1
Additional	D6	Have you ever used digital devices for assessment or intervention in life activities in your practice?	1=Yes; 2=No
Additional	D6_free	[If Yes] Please specify the devices and activities.	Free-text response
Additional	D7	Please provide your comments and impressions on this technology (MediaPipe-based analysis).	Free-text response

Note: All items from PE1 to AL3 were rated on a 5-point Likert scale (1 = strongly disagree to 5 = strongly agree).
